# Brain-wide human oscillatory local field potential activity during visual working memory

**DOI:** 10.1016/j.isci.2024.109130

**Published:** 2024-02-05

**Authors:** Balbir Singh, Zhengyang Wang, Leen M. Madiah, S. Elizabeth Gatti, Jenna N. Fulton, Graham W. Johnson, Rui Li, Benoit M. Dawant, Dario J. Englot, Sarah K. Bick, Shawniqua Williams Roberson, Christos Constantinidis

**Affiliations:** 1 Department of Biomedical Engineering, Vanderbilt University, Nashville, TN, USA; 2 Neuroscience Program, Vanderbilt University, Nashville, TN, USA; 3 Department of Neurology, Vanderbilt University Medical Center, Nashville, TN, USA; 4 Department of Neurological Surgery, Vanderbilt University Medical Center, Nashville, TN, USA; 5 Department of Electrical and Computer Engineering, Vanderbilt University, Nashville, TN, USA; 6 Department of Ophthalmology and Visual Sciences, Vanderbilt University Medical Center, Nashville, TN, USA

**Keywords:** Natural sciences, Biological sciences, Neuroscience, Systems neuroscience, Clinical neuroscience, Sensory neuroscience, Cognitive neuroscience

## Abstract

Oscillatory activity in the local field potential (LFP) is thought to be a marker of cognitive processes. To understand how it differentiates tasks and brain areas in humans, we recorded LFPs in 15 adults with intracranial depth electrodes, as they performed visual-spatial and shape working memory tasks. Stimulus appearance produced widespread, broad-band activation, including in occipital, parietal, temporal, insular, and prefrontal cortex, and the amygdala and hippocampus. Occipital cortex was characterized by most elevated power in the high-gamma (100–150 Hz) range during the visual stimulus presentation. The most consistent feature of the delay period was a systematic pattern of modulation in the beta frequency (16–40 Hz), which included a decrease in power of variable timing across areas, and rebound during the delay period. These results reveal the widespread nature of oscillatory activity across a broad brain network and region-specific signatures of oscillatory processes associated with visual working memory.

## Highlights

• We studied LFPs in human intracranial recordings during visual working memory
• Stimuli produced widespread, broad-band activation in an extensive brain network
• Occipital cortex was characterized by most elevated high-gamma power (100–150 Hz)
• Modulation in the beta frequency (16–40 Hz) characterized the delay period

## Graphical abstract

**Figure:**
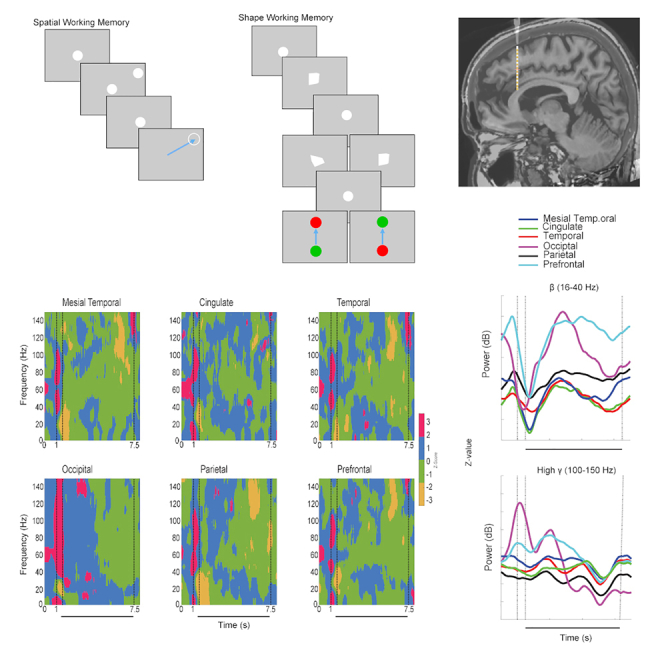

## Introduction

Working memory, the ability to hold and manipulate information in the mind for a short period of time, is central for many cognitive processes, including planning, problem-solving, and decision-making.^1^^,^^2^ Pathological conditions such as stroke, schizophrenia, and Alzheimer disease are associated with working memory impairments.^3^^,^^4^^,^^5^ For this reason, understanding the neural basis of working memory has been a long-standing question in cognitive neuroscience research.^6^ Neurophysiological experiments in non-human primates have identified neuronal activation of the prefrontal cortex as central for the maintenance of working memory.^7^ Persistent activity generated by the spiking of prefrontal neurons is also associated with distinct patterns of spectral power, identifiable in local field potential recordings.^8^^,^^9^

However, other lines of evidence, and particularly human functional imaging studies, suggest a much more widespread pattern of activation during working memory.^10^ Imaging studies have been successful in using multi-variate methods to decode the remembered visual stimuli from visual cortical area voxels.^11^ Although some neurophysiological studies have failed to detect persistent activity in the sensory cortices,^12^ it has been argued on theoretical grounds that no overall elevation of activity is necessary for cortical neurons to encode information, an idea often referred to as “activity-silent” mechanisms of working memory.^13^ Cellular and synaptic mechanisms have been implicated in the maintenance of working memory.^14^^,^^15^ Correspondingly, whether the contents of working memory are maintained in the prefrontal or in the sensory cortex has been a matter of debate.^16^^,^^17^

Part of the problem in reconciling these contrasting viewpoints is the use of different methodologies across human and animal studies.^18^ In recent years, analysis of intracranial recordings from human subjects have provided evidence for existence of persistent spiking activity during the maintenance of working memory^19^ and other properties of human brain activation during working memory.^20^^,^^21^^,^^22^^,^^23^ Analysis of non-human primate recordings has also begun to mirror methods of analysis inspired by human studies.^24^ However, differences in behavioral paradigms and methods of analyses persist and make comparison of findings from different models challenging. We were therefore motivated to collect and analyze local field potentials (LFPs) obtained from intracranial recordings in human subjects, using working memory behavioral paradigms and analysis methods that parallel those used in animal models. Our results provide a direct way to compare and reconcile findings in the respective research literatures of the two fields.

## Results

**Figure 1:**
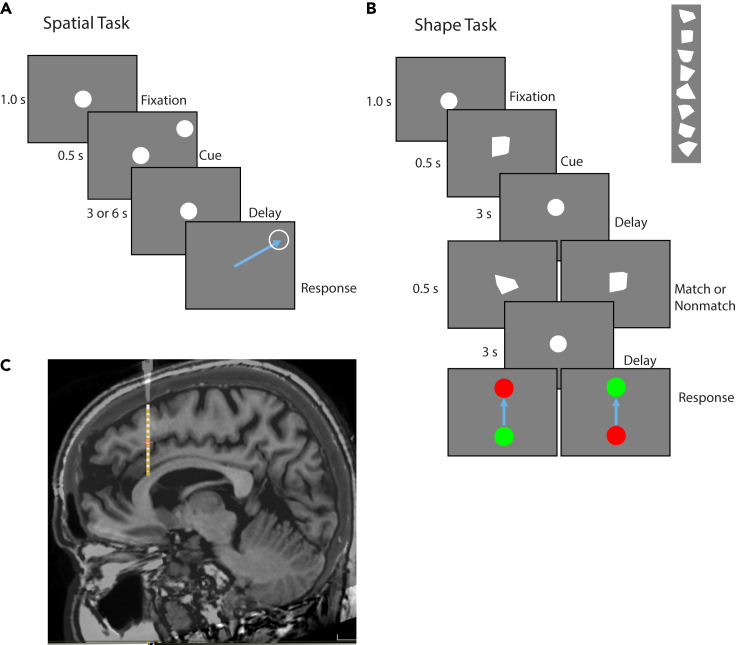
Behavioral tasks and recording methods (A) Spatial manual delayed response task. At the start of each trial, a circle appears in the center of the tablet screen, and the subject moves the stylus into the circle to initiate the trial. After 1 s, a second white dot appears (Cue) at a peripheral location for 0.5 s, after which only the center circle remains. After a delay period, the center circle disappears, and the subject needs to drag the stylus across the screen into the remembered location of the cue. (B) Shape delayed match-nonmatch task. At the start of each trial, a white circle appears in the center of the tablet screen, and the subject moves the stylus into the circle to initiate the trial. After a delay period, a white polygon replaces the center circle for 0.5 s (Cue), followed by the reappearance of the center circle. After a delay, a second convex polygon replaces the center circle for 0.5 s, followed by the reappearance of the center circle. After a second delay, the center circle disappears, and the subject needs to drag the stylus to either a green or red peripheral circle to indicate whether the two polygons were the same or not. (C) Example MRI scan of one patient, with electrode position, based on CT scan, superimposed, where LFP recording were made.

Fifteen participants were recruited for this study (Table 1). Each participant was implanted with an average of 12.5 ± 2.1 (mean ± SD) sEEG leads containing a total of 129.1 ± 35.9 active contacts (Figure 1C), with implant strategy and number of leads determined by clinical considerations. Two stimulus sets were presented (Figures 1A and 1B). One stimulus set varied the spatial location of a white circle (spatial task) and one involving different polygonal shapes (shape task). In the spatial task, the participants had to remember the location of the stimulus presented during the cue period and, after a 3-s or 6-s delay period, indicate where the stimulus appeared by dragging a pointer to the location of the stimulus. In the shape task, two stimuli were presented in sequence with an intervening delay period between them. The subjects needed to determine whether the two stimuli were identical or not and indicate their judgment by selecting a green or red choice target. Participants performed on average 38.4 ± 4.2 trials during the 3-s delay spatial task and 40.2 ± 9.4 trials during the 6-s delay spatial task. Of the 15 total participants, 12 performed 37.2 ± 5.0 trials in the shape task. Data collection lasted approximately 30 min, including instructions, practice runs, and pauses between tasks for rest.

**Table 1:** Patient demographic characteristics

Subj. number	Age (years)	Sex	Handedness	Seizure onset zone	Performance			
					Task 1	Task 2	Task 3	Mean
1	37	M	Right	Left medial temporal	95.5%	86.4%	N/A	90.9%
2	22	F	Right	Left medial temporal Left lateral temporal Left medial frontal Left orbitofrontal	93.9%	91.7%	N/A	92.8%
3	52	F	Right	Left medial temporal	66.7%	62.1%	N/A	64.4%
4	43	F	Right	Left temporal pole	33.3%	28.6%	63.0%	41.6%
5	29	F	Right	Left posterior temporal Left occipital	86.7%	69.7%	80.7%	79.0%
6	33	M	Left	Right medial temporal	87.5%	64.5%	80.7%	77.6%
7	30	F	Right	Right medial temporal Right lateral temporal	69.0%	76.5%	90.6%	78.7%
8	58	F	Right	Left medial temporal	74.3%	61.1%	61.3%	65.6%
9	43	F	Right	Left medial temporal	80.0%	63.0%	80.0%	74.3%
10	33	F	Right	Right orbitofrontal Right medial temporal Right basal temporal Right parietal	74.3%	68.6%	83.9%	74.6%
11	59	M	Right	Bilateral medial temporal	78.8%	67.7%	96.9%	81.1%
12	46	F	Right	Left medial frontal	88.2%	82.4%	90.0%	86.9%
13	49	F	Left	Bilateral temporal	77.8%	63.9%	72.4%	71.4%
14	49	F	Right	Left anterior temporal	94.4%	77.8%	90.3%	87.5%
15	46	F	Left	Cingulate	84.4%	73.3%	78.6%	78.8%
				Mean	79.0%	69.1%	80.7%	

Table reports age, sex, handedness, and seizure onset, as this was determined clinically, for study participants. Task performance in the 3-s spatial task, 6-s spatial task, and shape task (Task 1, 2, and 3, respectively) is shown for each patient. Mean across subjects and tasks is also shown.

LFP data were recorded from cortical and subcortical regions. To provide a concise overview of the patterns of LFP activity across the entire brain, we combined recordings into six regions, determined based on sample availability, anatomical proximity, and relative similarity of patterns of responses within region. These were defined as follows: (1) mesial temporal region, which included contacts from the amygdala, hippocampus, and mesial temporal cortex (n = 12 patients); (2) cingulate region, which included the anterior, mid, and posterior cingulate (n = 11); (3) lateral temporal region, which included contacts in the inferior temporal cortex and superior and middle temporal gyrus and insula (n = 14); (4) occipital region, which included any contacts in the occipital lobe (n = 3); (5) parietal region, which included contacts in the parietal lobe (typically in the posterior parietal cortex, n = 10); (6) prefrontal region, which included contacts in any subdivision of the prefrontal cortex (n = 13). A few contacts were not localized in any of these regions (e.g., were localized in subcortical structures—other than the hippocampus or amygdala). These were omitted from analyses presented here. Additionally, electrodes were excluded from data analysis if they were in the patient’s seizure onset zone based on review of seizures by the clinical team.

After preprocessing and artifact rejection, signals from a total of 1,024 contacts from 187 electrode shafts in the 3-s delay spatial task were available (197, 77, 350, 37, 110, and 253 contacts in the six regions named earlier, respectively). Additionally, 1,005 contacts from 145 electrodes were available in the 6s-delay spatial task (172, 79, 355, 37, 109, and 253 contacts in the six regions, respectively). Similarly, 854 contacts from 153 electrodes were available in the shape task (136, 46, 322, 37, 103, and 210 contacts in the six regions, respectively). Spectral power was computed in each trial and contact and then averaged within each region, for plotting purposes; we treated each electrode contact as one observation for statistical analysis.

### Signatures of spectral power for visual stimuli

**Figure 2:**
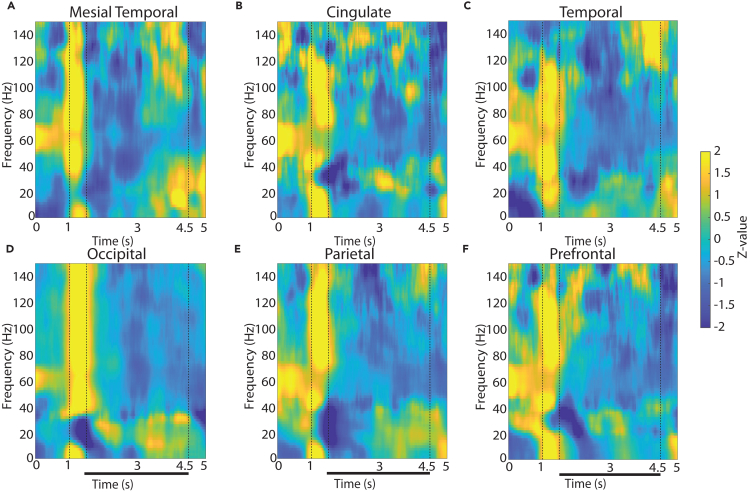
LFP power spectra across brain regions in the 3-s delay, spatial working memory task Average, induced power of the local field potential signal relative to baseline (computed in the intertrial interval) is shown for contacts grouped into six brain regions. Vertical lines indicate the time of stimulus presentation (1–1.5 s) and the beginning of the response period (4.5 s). Horizontal bar indicates the delay period of the task over which stimuli needed to be maintained in working memory. (A) Mesial temporal regions (amygdala, hippocampus, mesial temporal cortex). n = 12 subjects; n = 197 electrode contacts; n = 3,435 trials. (B) Cingulate regions (anterior cingulate, posterior cingulate). n = 11 subjects; n = 77 contacts; n = 1,805 trials. (C) Temporal regions (inferior temporal cortex, superior and middle temporal gyrus, insula). n = 14 subjects; n = 350 contacts; n = 6,931 trials. (D) Occipital regions. n = 3 subjects; n = 37 contacts; n = 967 trials. (E) Parietal regions. n = 10 subjects; n = 110 contacts; n = 2,837 trials. (F) Prefrontal regions. n = 13 subjects; n = 253 contacts; n = 5,840 trials.

Our analysis first focused on how the visual stimuli themselves induced LFP power across spectral bands and areas. We therefore determined the time course of induced LFP power during the trial. Mean power in six different brain regions is shown for the two versions (3-s and 6-s delay) of the spatial working memory task in Figures 2 and S1, respectively. The most salient finding of this analysis was a broadband signal during the appearance of the stimulus. This was present not only in the visual areas of the occipital lobe but also in the temporal, parietal, and prefrontal association cortices and even in areas not traditionally linked to strong sensory responses, such as the cingulate and mesial temporal lobe (see cue presentation interval between the dotted lines at 1 and 1.5 s in Figure 2). The broadband power elevation remained prominent when plotting the power spectrum in a logarithmic frequency scale (Figure S2).

**Figure 3:**
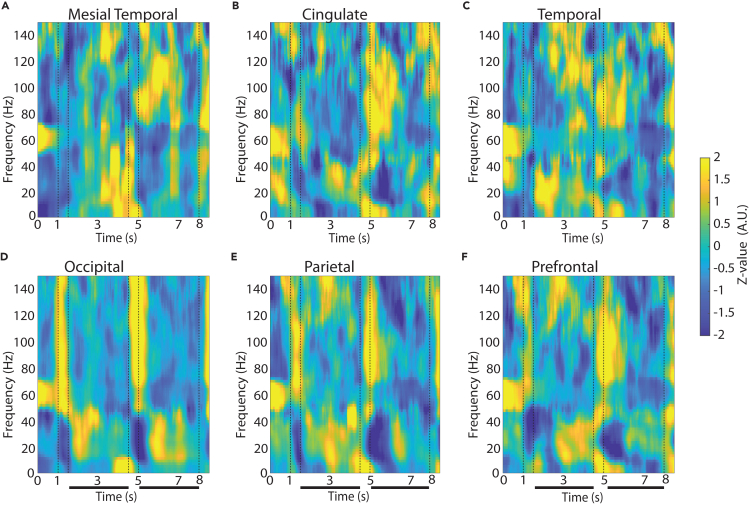
LFP power spectra across brain regions in the shape, match-nonmatch working memory task Average, induced power of the local field potential as in Figure 3, now plotted for the shape match-nonmatch, working memory task. Vertical lines indicate the time of the two stimulus presentations (1–1.5 s and 4.5–5) and the beginning of the response period (8 s). Horizontal bars indicate the delay periods of the task over which stimuli needed to be maintained in working memory. (A) Mesial temporal regions (amygdala, hippocampus, mesial temporal cortex). n = 9 subjects; n = 136 electrode contacts; n = 2,214 trials. (B) Cingulate regions (anterior cingulate, posterior cingulate). n = 8 subjects; n = 46 contacts; n = 746 trials. (C) Lateral temporal regions (inferior temporal cortex, superior and middle temporal gyrus and insula). n = 10 subjects; n = 322 contacts; n = 6,022 trials. (D) Occipital regions. n = 3 subjects; n = 37 contacts; n = 843 trials. (E) Parietal regions. n = 9 subjects; n = 103 contacts; n = 2,339 trials. (F) Prefrontal regions. n = 10 subjects; n = 210 contacts; n = 4,116 trials.

The effect of stimulus presentation diminished for the shape working memory task (Figure 3), which involved presentation of stimuli over the fovea, replacing the pre-existing fixation point. Only in this latter task, the presentation of the visual stimulus elicited preferential broadband activation in the occipital cortex, although an LFP power transient was also evident in parietal and prefrontal cortex (Figure 3).

The most differentiating effect of the cue appearance across regions was the extent of the increase in high-gamma (100–150 Hz) power, which is likely most closely tied to neuronal spiking.^20^ To identify differences between regions and conditions, we calculated power in each task epoch, after subtracting at each frequency the baseline power, which was computed in the inter-trial interval. We averaged this power for all trials obtained from each contact in a given electrode and treated it as a single observation (though treating multiple electrode contacts from the same patient, as distinct). We adopted a mixed-effects model, with brain regions as a fixed factor and added a random effect term for the participants, to avoid the confound of unequally represented electrode contacts in regions across participants. Averaging power in this range across the entire cue presentation period revealed a highly significant difference between the six regions in the 3-s spatial working memory task (F_5,1096_ = 19.98, p = 2.2E-16, effect size: η^2^ = 0.09). The occipital region exhibited the highest power in the high-gamma range compared with all other regions (Tukey post-hoc test, evaluated at the α = 0.05 significance level). High-gamma power was also significantly different between areas in the 6-s delay version of the spatial task (F_5,998_ = 19.06, p = 2.2E-16, effect size: η^2^ = 0.09) and in the shape working memory task (F_5,840_ = 2.78, p = 0.02, effect size: η^2^ = 0.02). The occipital region again exhibited significantly higher power in high-gamma frequency compared with all other regions (Tukey post-hoc test, evaluated at the α = 0.05 significance level).

### Delay-period LFP power

**Figure 4:**
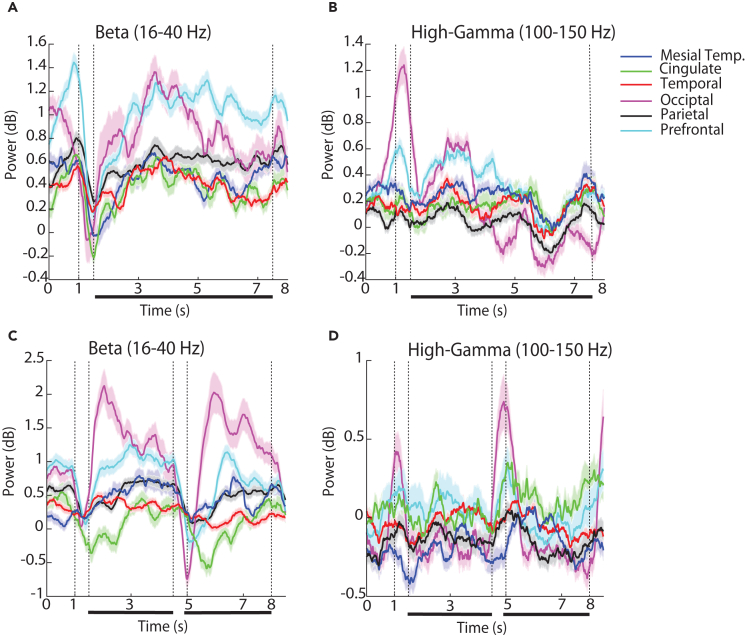
Time course of beta and high-gamma power (A) Time resolved induced LFP power in the beta frequency range (16–40 Hz) for the 6-s spatial working memory task, in each of the six brain regions identified. Data are represented as mean (solid line) and standard error of the mean (shaded area). (B) LFP power in the high-gamma range (100–150 Hz). (C) Similarly, time resolved induced LFP power in the beta frequency range (16–40 Hz) for the shape working memory task, in each of the six brain regions identified. (D) LFP power in the high-gamma range (100–150 Hz).

We next tested whether the high-gamma power differences observed in the cue presentation interval carried over to the delay period. This was generally not the case. High-gamma power in the occipital cortex essentially returned to the pre-fixation baseline during the delay interval (Figure 4B). Instead, substantial differences during the delay interval were mostly evident in the high-beta frequency band (16–40 Hz). Shortly after the cue appearance, beta power decreased with a timing of the beta frequency trough that differed between areas. In the 3-s delay spatial task, it reached a minimum at the occipital cortex after the stimulus offset, at the parietal cortex 150 ms later, and the prefrontal cortex another 650 ms later (Figure 2). A trough was also evident in the 6-s spatial task (Figure 4A). Averaging between power across the delay period revealed systematic differences between regions in the 3-s and 6-s spatial task (Figure S3, F_5,1006_ = 3.24, p = 0.007, effect size: η^2^ = 0.02 and F_5,995_ = 11.37, p = 1.1E-10, effect size: η^2^ = 0.05 for the two versions, respectively). In the shape task, a beta power trough was evident around each of the two stimulus presentations (Figure 4C), which rebounded earlier the baseline during the delay interval, particularly for the occipital and prefrontal cortex. In this case, too, beta power differed systematically between regions during the first delay period (F_5,840_ = 12.03, p = 2.88E-11, effect size: η^2^ = 0.07), with the occipital region exhibiting the greatest amplitude (Figure 4C). The prefrontal cortex was the area with the highest power throughout the delay period of the task (Figure 4D).

### Task differences

**Figure 5:**
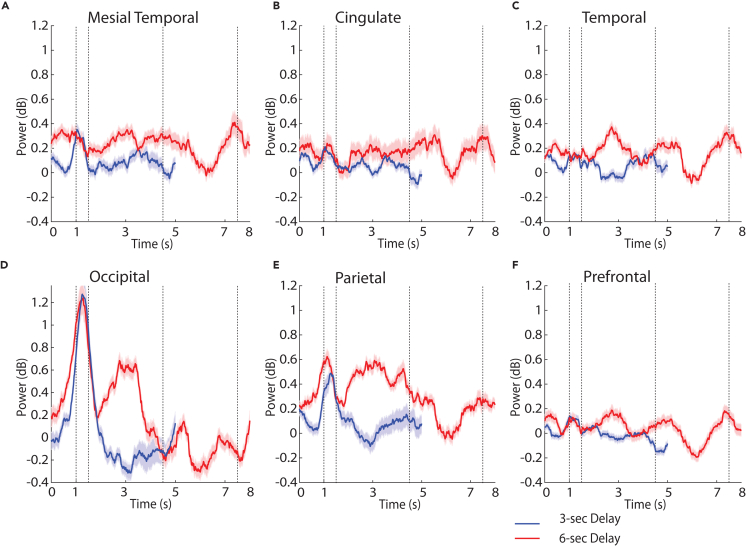
High-gamma power for different delay durations Time resolved induced LFP power in the high-gamma range (100–150 Hz) for the two versions of the spatial working memory task, involving 3-s and 6-s delay periods. (A) Mesial temporal regions. (B) Cingulate regions. (C) Temporal regions. (D) Occipital regions. (E) Parietal regions. (F) Prefrontal regions.

The two versions of the spatial task imposed different difficulty in terms of their working memory requirement (3 vs. 6 s of working memory maintenance period). This was also reflected in the performance of subjects in the two versions of the tasks, which was lower in the 6-s delay variant: 79% vs. 69% for Task 1 and Task 2, respectively, in Table 1, a significant difference (two-tailed paired t test, t_14_ = 4.93, p = 0.0004). This difference resulted in differences in spectral power across regions, specifically in the high-gamma range (Figure 5). Here, we adopted a mixed-effects model, with fixed factors, the regions, and the 3-s/6-s version of the spatial task and added a random effect term for participants. We considered the first 3 s of the delay period in the two task versions to compare power. High-gamma power was higher in the 6-s delay version of the task (F_1,2004_ = 54.86, p = 1.89E-13, effect size: η^2^ = 0.03 for main effect of task). We also found an interaction between regions and versions of the spatial task (F_5,2004_ = 4.32, p = 0.0006, effect size: η^2^ = 0.01). The parietal region (Figure 5) exhibited significantly higher power compared with other regions (F_5,2007_ = 4.21, p = 0.0008, effect size: η^2^ = 0.01, Tukey post-hoc test, evaluated at the α = 0.05 significance level). It was also notable that the elevated high-gamma power in the 6-s version of the task was already present in the fixation period, prior to the appearance of the stimulus or the delay period. Because the tasks were presented in blocks, the result suggests that the participants’ level of effort or expectations about the task modulated systematically spectral power. Such differences were mostly absent in the beta frequency range (Figure S3).

We also considered high-gamma power in the delay period recorded from the same electrode contacts that were available in both 3-s and 6-s version of the spatial task (Figure S4). Because contacts were perfectly balanced across subjects in this analysis, we relied on a repeated measures ANOVA model. This analysis revealed that delay-period high-gamma power differed systematically between the two tasks (F_1,989_ = 9.72, p = 3.4E-11) with power in the parietal and occipital areas exhibiting the greatest difference between tasks.

The shape task differed in the type of stimuli that needed to be maintained in memory (shapes rather than spatial locations), the part of the visual field where the stimuli appeared (central vs. peripheral), and also in the nature of the task (requiring a judgment on whether the second stimulus was match or nonmatch and preparation of a motor response based on a color rule). As already mentioned, the broadband increase in power that was observed across all areas when peripheral visual stimuli were presented in the spatial working memory task was more limited to the occipital cortex in this case (Figure S1). To perform the shape task, it was still essential for subjects to maintain the identity of the stimulus in memory at least during the first delay period. Thus, we examined how the high-gamma differed between regions as the subjects were engaged in this function (Figures 3 and 4). High-gamma power differed even less so between regions in the shape task (Figure 4D, F_5,840_ = 1.11, p = 0.35) than in the spatial task.

We next compared high-gamma power between the spatial and shape task. Here, we adopted a mixed-effects model, with fixed factors, the regions and tasks, and added a random effect term for participants. We first considered the average power of the first 3 s in the delay period of the 6-s version of the spatial task and the first delay period of the shape task. High-gamma power was higher overall in the spatial task (F_1,1837_ = 91.3, p = 2E-16, effect size: η^2^ = 0.05 for main effect of task). This analysis revealed no main effect of differences between regions (F_5,1837_ = 0.65, p = 0.66, effect size: η^2^ = 0.001). An interaction between regions and tasks (F_5,1834_ = 2.84, p = 0.01, effect size: η^2^ = 0.007) was present, however, evidenced by the different order of high-gamma power between regions in Figures 4B and 4D. Prefrontal high-gamma power was consistently high compared with other areas, particularly early in the delay period, in both tasks but high-gamma power of other areas, such as cingulate cortex, was more pronounced in the shape task, and high-gamma power of parietal cortex was more pronounced in the spatial task.

To confirm that these findings were specific to the nature of the task executed, we also compared high-gamma power between the 3-s version of the spatial task and the shape task. This analysis confirmed that high-gamma power was higher overall in the spatial task (F_1,1856_ = 17.74, p = 2.65E-5, effect size: η^2^ = 0.009 for main effect of task). An interaction between regions and tasks was also evidenced (F_5,1853_ = 2.68, p = 0.02, effect size: η^2^ = 0.007).

## Discussion

Our study revealed patterns of oscillatory brain activity during the execution of visual working memory tasks including widespread and region-specific patterns of activity visible in the LFP. Firstly, we found that the task produced a broadband LFP signal in a brain-wide network, including in areas not traditionally thought to be engaged in visual processing, such as the mesial temporal and cingulate cortex. Secondly, we found that high-gamma power (100–150 Hz), which is likely to be associated with neuronal spiking,^25^ was indeed most pronounced in the occipital cortex during the visual stimulus presentation, but this activation did not persist in the delay period, in contradiction to theories supporting maintenance of working memory in the sensory cortex. Thirdly, we found that beta power (16–40 Hz) was most characteristic of the delay (working memory maintenance) period, which however had a nonmonotonic modulation; this included a transient trough of power after the stimulus appearance that rebounded later during the delay period, with the prefrontal cortex being most pronounced in this respect. This pattern of beta power was remarkably similar to the one observed in the monkey prefrontal cortex as subjects performed the identical working memory task but not during passive viewing of the same stimuli, prior to training.^9^

Our results indicate widespread activation of cortex during visual working memory tasks, in partial agreement with prior results from human imaging studies^11^^,^^26^^,^^27^^,^^28^^,^^29^^,^^30^ and as predicted by modeling studies analyzing monkey results.^31^ They also suggest, however, that volume conduction signals such as the LFP (and BOLD) undergo nonmonotonic modulation between the stimulus presentation and working memory maintenance interval, making interpretation of such a signal difficult. Finally, some properties of the prefrontal cortex appeared to be unique in the critical interval of working memory maintenance, in agreement with both human^32^ and animal studies.^33^ These results are also consistent with hierarchical processing of visual information and support the idea that memory may travel along the sensory processing stream of the utilized sensory modality.^34^

Our task design made it possible to directly compare our findings with neurophysiological results obtained in non-human primate studies, which have the ability to record from large numbers of neurons in very localized areas.^35^ Our findings were also directly related to imaging studies, which have simultaneous access to all brain regions, but with lower temporal and spatial resolution.^32^ Our approach therefore offers a path to directly bridge these literatures.

### Regional localization of working memory

The locus of working memory maintenance in the brain has been a matter of debate in recent years. Neurons in the prefrontal cortex and areas connected with it generate persistent discharges tuned for stimulus properties during the delay intervals of working memory tasks, in animal^36^ and human intracranial recordings.^19^ Computational models typically simulate persistent activity in neural networks with recurrent connections between units with similar tuning for stimuli,^37^ which capture working memory behavior very well, particularly in spatial working memory tasks.^38^ Based on these results, it has been postulated that the prefrontal cortex plays the primary role in the maintenance of working memory by virtue of generation of persistent spiking activity.^17^

However, this idea has been challenged. Human imaging studies have applied multi-variate pattern analysis (MVPA), examining the simultaneous pattern of activation of multiple voxels to different task conditions,^39^ in order to successfully decode working memory content from the primary^11^^,^^26^^,^^27^ and extrastriate visual cortex.^28^^,^^29^^,^^30^ On these grounds, it has been suggested that the prefrontal cortex may play a supervisory or control role in working memory, “highlighting” the locations of stimuli held in memory, whereas the contents of working memory are maintained in sensory cortex.^16^

This is not to say, however, that models of prefrontal persistent activity are inconsistent with our findings. It is understood that a distributed network of cortical and subcortical areas generates persistent activity during working memory.^10^^,^^40^ The prefrontal cortex is essential for the ability of the network to maintain persistent discharges, by virtue of the biophysical properties of its neurons and pattern of connectivity.^12^^,^^31^ In our results, high-gamma power was consistently elevated in the prefrontal cortex during the delay period across tasks (Figures 4B and 4D), although this difference was quantitative rather than qualitative in comparison with other areas, and high-gamma power may be an imprecise index of working memory (as discussed in the next section). The presence of oscillatory processes in other brain areas was also revealing. Mesial temporal structures, such as the hippocampus and amygdala, play well-understood roles in long-term memory.^41^^,^^42^ More recent studies, however, suggest engagement during working memory processes, as well.^19^^,^^21^^,^^43^ Our results confirmed these findings and suggested modulation of oscillatory processes by the working memory tasks.

### Basis of working memory in neural activity

As discussed, models of working memory informed mostly by animal studies have identified persistent discharges generated in the prefrontal cortex and other areas as the critical neural correlate of working memory.^17^ Such persistent spiking generation is most often associated with gamma frequency oscillations in the LFP.^44^ Some recent working memory models have emphasized the rhythmicity of spiking discharges themselves and posited that bursting in the gamma frequency range is the critical variable that tracks stimulus information maintenance in working memory.^45^^,^^46^ Human intracranial recordings, too, reveal rhythmicity in the gamma band during working memory tasks.^20^^,^^47^^,^^48^ Critical task parameters, such as working memory load, have been shown to modulate neural oscillations.^49^^,^^50^ For this reason, gamma power is considered a marker of task-related activation.^51^

However, gamma frequency oscillation in the LFP is at best an imprecise index of neural activity mediating working memory. The LFP represents summation of ionic currents in a cortical volume, in the order of 0.1–0.2 mm and are driven by both spiking and synaptic events, e.g., postsynaptic potentials propagated from distant areas that fail to generate action potentials in the area where the recording takes place.^52^^,^^53^ During presentation of stimuli, correlated bottom-up inputs can serve to synchronize population neuronal spiking, and phases of synchronized excitation by pyramidal neurons followed by inhibition by interneurons can thus produce rhythmicity specifically in the gamma frequency range.^54^ Less precisely timed or correlated inputs may fail to generate gamma oscillations, and indeed recent animal studies have suggested more prominent changes in the beta rather than gamma frequency range after learning to perform working memory tasks.^8^^,^^9^ Similarly, a recent human study of activation patterns in auditory working memory with intracranial recordings demonstrated that frontal and temporal regions with high decoding accuracy were not accompanied by significant increases in gamma power.^55^

Beta frequency oscillations are thought to represent a signature of activity during top-down processes, which is disrupted by appearance of exogenous, sensory stimuli.^56^^,^^57^^,^^58^ Beta oscillations are readily detectible in other extracellular field recordings (such as EEG or MEG) and are also a reliable marker of underlying cognitive processes impacting neural circuit interactions, just as gamma oscillations are,^50^^,^^59^ including working memory and top-down control.^60^^,^^61^ Consistent with the aforementioned animal studies,^8^^,^^9^ in our study, decrement of beta oscillations was detected during the task execution, and differed systematically between areas, at least around the time of stimulus presentations and early in the delay period of the task.

### Limitations of the study

Some limitations apply to our study. First, the number of subjects was relatively small, as was the number of trials they completed. The limited number of subjects affected sampling of some areas disproportionately and particularly the occipital cortex. The position of electrodes in intracranial recordings is dictated by clinical need, and recordings from visual cortex (and other sensory fields) are rare. Our results, from the occipital cortex in particular, ought to be interpreted with caution.

Although our analysis focused on trials completed correctly, it is important to note that epilepsy patients are known to suffer from cognitive deficiencies, including in frontal lobe function and working memory.^62^ In this respect, patterns of brain activity we describe here may deviate systematically from those of healthy participants. Some patients achieved low performance in the task (for example, patient 4 in Table 1), although many of the errors were due to accuracy and timing of responses rather than failure of working memory *per se*, and for this reason we opted not to exclude the (correctly completed) trials of any patient from our analysis.

Additionally, our results relied entirely on LFPs. Although providing an informative reflection of neural activity, LFP power does not map strictly onto spiking activity.^53^ Results from combined methodologies, including neuron recordings from large populations of isolated neurons in humans and animal models, and combined single-neuron and LFP analysis in the near future are making it possible to study in detail the role of areas and patterns of neuronal activity in working memory.

## STAR★Methods

### Key resources table

**Table:** 

REAGENT or RESOURCE	SOURCE	IDENTIFIER
**Deposited data**		

Code and Data for analysis and Figures	This paper	https://data.mendeley.com/preview/ynd4yn4wfm?a=1b4d27c6-85cd-471d-a6bf-074472c7a39b

**Software and algorithms**		

MATLAB	MathWorks	R2015-2022a
Chronux	http://chronux.org/	v2.12
FieldTrip	http://www.fieldtriptoolbox.org/	Fieldtrip-20190911
R package and Rstudio	https://support.posit.co/hc/en-us/articles/200486488-Developing-Packages-with-the-RStudio-IDE	ImerTest R package
Psychophysics Toolbox		v3
CRAnial Vault Explorer (CRAVE)	https://doi.org/10.1016/j.media.2010.07.009	CRAVE
FreeSurfer	http://surfer.nmr.mgh. harvard.edu	v7.2

**Other**		

Natus clinical data acquisition system	Middleton, WI	Natus
SEEG Electrodes	PMT corporation, Chanhassen, MN	0.8 mm in diameter and containing 8-16 contacts, spaced between 2.5- 4.3 mm center-to-center
Portable Tablet	Microsoft Surface, Redmond WA	26.0 cm-by-17.3 cm display and stylus

### Resource availability

#### Lead contact

Further information and requests for resources and reagents should be directed to and will be fulfilled by the Lead Contact, Dr. Christos Constantinidis (Christos.Constantinidis.1@vanderbilt.edu).

#### Materials availability

This study did not generate new unique reagents.

#### Data and code availability

• Data used for the analysis and figures will be deposited at Mendeley.com and be made publicly available as of the date of publication. DOIs are listed in key resources table.
• This paper does not report original code.
• Any additional information required to reanalyze the data reported in this paper is available from the lead contact upon request.

### Experimental model and study participant details

Fifteen epilepsy patients, 12 female, 3 male;15 white, 1 Hispanic; ranging from 22-59 years old (mean ± std; 41.9 ± 10.9) participated in the visual working memory study. These were patients with medically intractable epilepsy who had stereo electro-encephalography (sEEG) electrodes implanted to localize their seizure focus. Electrodes were implanted under general anesthesia with implant locations determined on an individual subject basis by clinical considerations related to hypotheses for site of seizure onset. Following surgery, patients were admitted to the epilepsy monitoring unit and antiepileptic medications gradually weaned while patients were continuously monitored for seizures. For our study, we collected LFPs from the contacts of sEEG electrodes using the Natus clinical data acquisition system (Natus, Middleton, WI). All subjects gave informed consent for this study, which was approved by the Institutional Review Board of the Vanderbilt University Medical Center (Nashville, TN, IRB #211037).

### Method details

#### Behavioral tasks

Participants performed visual working memory tasks using a portable tablet with a 26.0 cm-by-17.3 cm display and stylus (Microsoft Surface, Redmond WA) in their hospital rooms, while LFPs were continuously recorded from sEEG electrodes. Task control was achieved in MATLAB 2021 (MathWorks, Natick, MA) using the Psychophysics Toolbox, Version 3.^63^ At the beginning of each task epoch, the serial port triggers an Arduino Leonardo to send a TTL pulse as timestamps to the trigger channel of the EEG amplifier.^64^

Variants of a manual delayed response task (spatial task) and a shape match-nonmatch task (shape task) were used. In the spatial task (Figure 1A), a circle 2 cm in diameter appeared at the start of each trial in the center of the tablet screen and the subject moved the stylus into the circle to initiate the trial. After a 1 s “fixation” period, a second white circle of the same size appeared (cue) at a peripheral location 6.1 cm away from the center of the display for 0.5 s, after which only the center circle remained. This is followed by a 3 s or 6 s (delay) interval where, again, only the fixation point was displayed. After the delay period, the center circle disappeared, and the subject needed to drag the stylus across the screen into within an invisible circle 2.4 cm in diameter centered at the remembered location of the cue. The stylus needed to enter the invisible circle within 2 s and stay inside for 0.5 s for the trial to be considered correct. A TTL pulse marked the beginning of each task event. Delay durations were blocked; trials with 3 s delays were performed first, and then the 6 s delay trials were presented. Cue locations in a block were evenly distributed across 360 degrees starting at 0 degrees. A typical block had 36 cue locations, each of which appeared once. Cue locations in completed trials were not repeated within a block.

In the shape task (Figure 1B), at the start of each trial, a circle appeared in the center of the tablet screen, and the subject moved the stylus into the circle to initiate the trial. The circle remained visible for a 1 s fixation period. Then a stimulus was presented for 0.5 s, comprising a white, convex polygon (cue), replacing the center circle. This is followed by a 3 s (delay) interval where, again, only the white circle was displayed. A second stimulus (sample) was subsequently shown for another 0.5 s, followed by a second 3 s delay period. At the end of the second delay period, the center circle disappeared, and two colored circles appeared at the top and bottom of the screen. The subject then needed to drag the stylus across the screen into either the green circle or red circle to indicate whether the two polygons were the same (match) or not (nonmatch), respectively. The polygons used had 3 to 6 vertices, did not extend beyond the extend of the center circle and no polygon was a rotated version of another. The red and green circles’ placement was randomly switched between the top and bottom locations on each trial. A total of eight shapes were used, followed by either a top and bottom location in match or nonmatch to create 32 trials. The circle sizes and the response window duration in the shape task were the same as those in the spatial task.

Before participants performed the full block of the visual working memory tasks, a member of the experimenter team explained and demonstrated the procedure. The participants then practiced a few trials to ensure they understand the experimental protocol before the block began.

#### Electrode localization

Participants were implanted with multiple electrode shafts of 0.8 mm in diameter and containing 8-16 contacts, spaced between 2.5- 4.3 mm center-to-center (PMT corporation, Chanhassen, MN). We identified the location of all contacts in each individual’s brain by superimposing the pre-surgical MRI and postsurgical CT images using the CRAnial Vault Explorer (CRAVE) software^65^ and the FreeSurfer software package (http://surfer.nmr.mgh.harvard.edu). These software tools allowed us to determine coordinates of each contact, and to identify the anatomical location of each contact according to the Desikan-Killiani Atlas^66^ using FreeSurfer’s cortical parcellation and subcortical segmentation procedure.^67^^,^^68^ White matter contacts were excluded from analysis. Electrode contact localizations obtained in this manner were additionally verified by an epileptologist (SWR) based on visual inspection of the co-registered MRI, postsurgical CT and baseline EEG timeseries.

#### LFP recording, preprocessing and signal analysis

Local field potentials were recorded from the implanted sEEG electrodes and sampled at 512 Hz. Reference electrodes were placed on the scalp, at a position that differed in each subject, most commonly placed over Cz, Pz and between Cz-Pz. If these areas were not available due to surgical implant location, then it was placed over Fz. Task events were synchronized with the LFP recording with a TTL pulse that was generated by the tablet and was recorded in the data acquisition system. Task epochs (e.g., fixation, stimulus presentation, delay period) were aligned to different TTL pulses. LFP recordings were preprocessed by using custom MATLAB code in MATLAB R2022b (MathWorks) and the FieldTrip toolbox.^69^ A bandpass filter between 0.5-200 Hz with a zero-phase sixth-order Butterworth filter were used on single-trial LFP traces. To remove 60 Hz powerline noise and its harmonics, an infinite impulse response (IIR) Butterworth filter was applied. Further, single-trial LFP traces underwent manual inspection for artifact rejection. Electrodes were excluded from data analysis if they were not in gray matter or were determined to be within the patient’s seizure onset zone based on review of seizures by the clinical team and confirmed by a clinical epileptologist (SWR).

The Chronux package^70^ was used for time-frequency analysis. We used a multi-taper method to perform a power spectrum analysis of LFPs. The spectrogram of each single trial between 0.5 and 150 Hz was computed with 8 tapers in 500 ms time windows; the spectrograms were estimated with a temporal resolution of 2 ms. We also used the mean filter corresponding to 2 Hz and 2 ms for smoothing the spectrogram of each single trial. In all our analyses, we relied on induced power of the LFP, which is computed by first performing a power computation in each trial and then averaging power across trials. Induced power thus determines power at specific frequencies that may not necessarily be synchronized with specific task events across trials. Power was expressed relative to the mean power recorded during the inter-trial interval. We constructed time-resolved plots (spectrograms) by dividing the power of the signal by the mean inter-trial interval power at each frequency (which is equivalent to subtracting the baseline power in logarithmic, dB, scale). We then standardized the algorithmic power on the temporal profile at each frequency.

### Quantification and statistical analysis

Statistical testing of differences between conditions was performed in the following fashion, using only trials that participants completed correctly. First, we calculated power across an entire task epoch: e.g., cue presentation or delay period. Secondly, we averaged power values in these epochs from all available trials of every electrode site, essentially treating each electrode contact as one observation. We then constructed a 1-way or 2-way mixed-effects linear model with fixed-effects terms representing the region, and task and random effects term for each participant as follows:

power∼region+(1|participant)

power∼region∗task+(1|participant)

In every case, the analysis was performed for the beta and high-gamma frequency band, defined as 16-40 Hz and 100-150 Hz, respectively, based on prior studies of working memory.^8^^,^^20^^,^^46^ These models were implemented in the R computational environment using the lmer R package and Rstudio. F-statistics and p-values were calculated with Satterthwaite’s or Kenward-Roger’s method for degrees-of-freedom determination. F-statistics were calculated based on the glme model (ImerTest R package). The effect size was determined by the partial eta-squared ($\eta^{2}$). Post-hoc pairwise comparisons on any significant main effects were performed with Tukey’s method.
