# Supplementary material for: Brain-wide human oscillatory local field potential activity during visual working memory
Source: iScience. 2024 Feb 5;27(3):109130. doi: 10.1016/j.isci.2024.109130 (PMC10877957; doi:10.1016/j.isci.2024.109130)
Supplement: Document S1. Figures S1–S4 [file mmc1.pdf]

iScience, Volume *27*

Supplemental information

Brain-wide human oscillatory local field potential activity during visual working memory

Balbir Singh, Zhengyang Wang, Leen M. Madiah, S. Elizabeth Gatti, Jenna N. Fulton, Graham W. Johnson, Rui Li, Benoit M. Dawant, Dario J. Englot, Sarah K. Bick, Shawniqua Williams Roberson, and Christos Constantinidis

Supplementary Material for:

Brain wide human oscillatory local field potential activity during visual working memory

Balbir Singh[1], Zhengyang Wang[2], Leen M. Madiah[1], S. Elizabeth Gatti[1], Jenna N. Fulton[3], Graham W. Johnson[4], Rui Li[5], Benoit M. Dawant[5], Dario J. Englot[1,4], Sarah K. Bick[1,4], Shawniqua Williams Roberson[1,3], Christos Constantinidis[1,2,6]

1. Department of Biomedical Engineering, Vanderbilt University
2. Neuroscience Program, Vanderbilt University
3. Department of Neurology, Vanderbilt University Medical Center
4. Department of Neurological Surgery, Vanderbilt University Medical Center
5. Department of Electrical and Computer Engineering, Vanderbilt University
6. Department of Ophthalmology and Visual Sciences, Vanderbilt University Medical Center

Keywords: LFP, intracranial recordings, working memory

Lead Contact / Corresponding Author:
Christos.Constantinidis.1@vanderbilt.edu

This PDF file includes:
Supplementary Figure 1-4

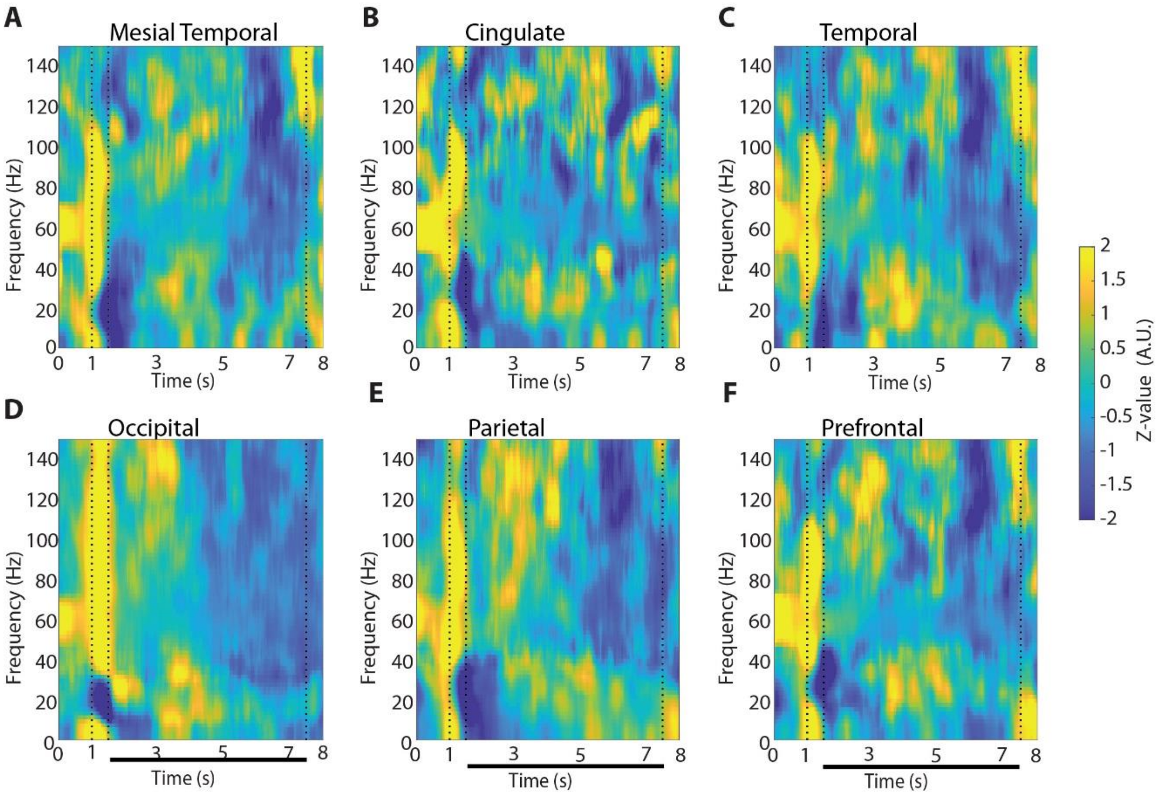

Figure S1, related to Figure 2. LFP Power spectra across brain regions in the 6-s delay, spatial working memory task. Average, induced power of the local field potential as in Figure 3, now plotted for the 6-s delay version of the spatial working memory task. Vertical lines indicate the time of stimulus presentation (1-1.5 s) and the beginning of the response period (7.5 s). Horizontal bar indicates the delay period of the task over which stimuli needed to be maintained in working memory. **A.** Mesial Temporal regions (Amygdala, Hippocampus, Mesial Temporal Cortex) n= 12 subjects; n=172 electrode contacts, n=2638 trials. **B.** Cingulate regions (Anterior Cingulate; Posterior Cingulate) n= 11 subjects; n=79 contacts, n=1553 trials. **C.** Temporal regions (Inferior Temporal Cortex) n= 14 subjects; n=355 contacts, n=5715 trials. **D.** Occipital regions n= 3 subjects; n=37 contacts, n=587 trials. **E.** Parietal regions n= 10 subjects; n=109 contacts, n=2071 trials. **F.** Prefrontal regions n= 13 subjects; n=253 contacts, n=4820 trials.

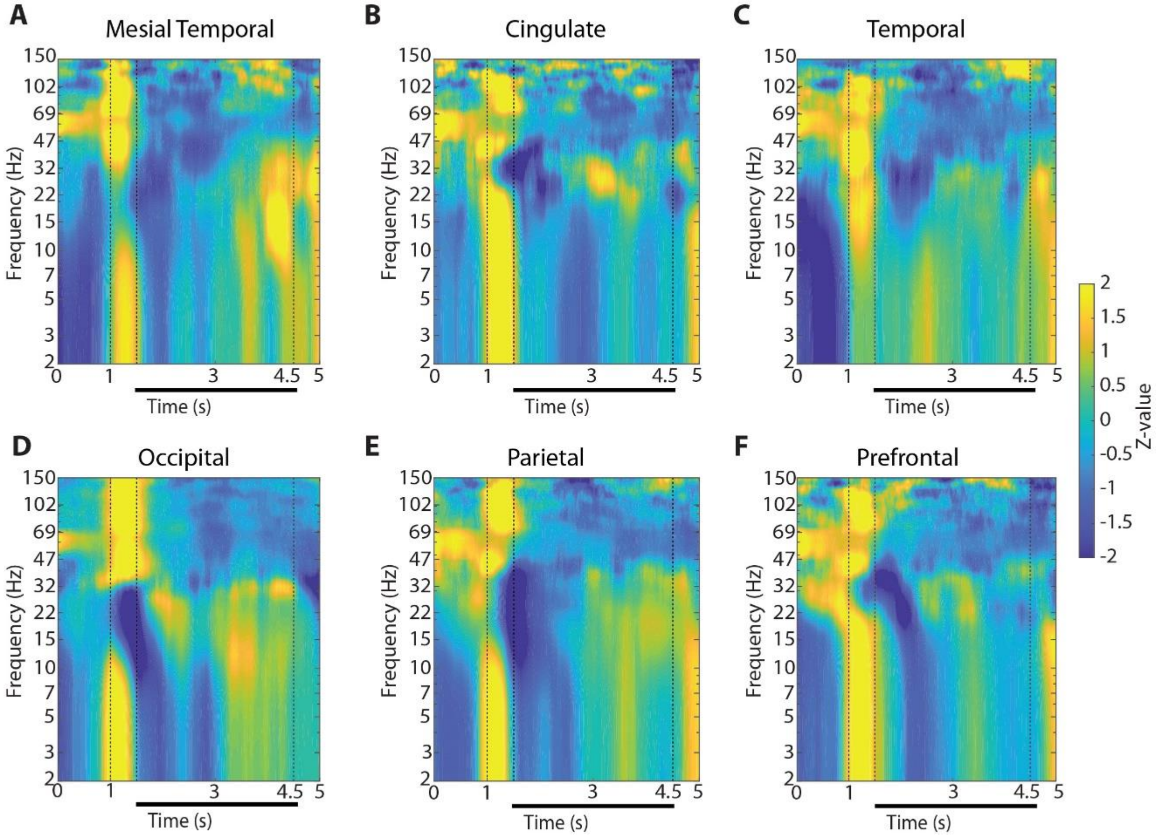

Figure S2, related to Figure 2. LFP Power spectra in the 3-s delay, in logarithmic frequency scale. Same dataset of average, induced power of the local field potential signal relative to baseline as in Figure 2, plotted with a y-axis in a logarithmic scale.

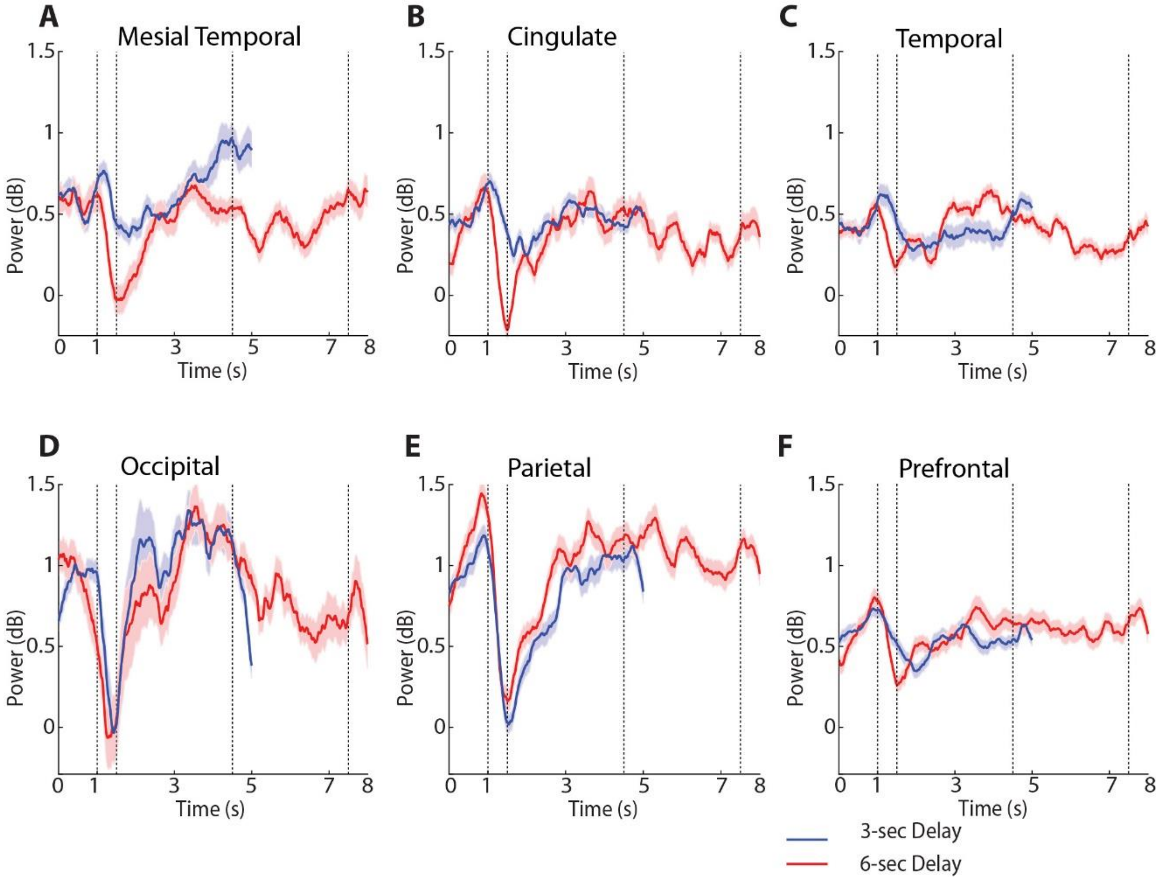

Figure S3, related to Figure 5. Beta power for different delay durations. Time resolved induced LFP power in the beta range (16-40 Hz) for the two versions of the spatial working memory task, involving 3 s and 6 s delay periods. A. Mesial Temporal regions. B. Cingulate regions. C. Temporal regions. D. Occipital regions. E. Parietal Regions. F. Prefrontal regions.

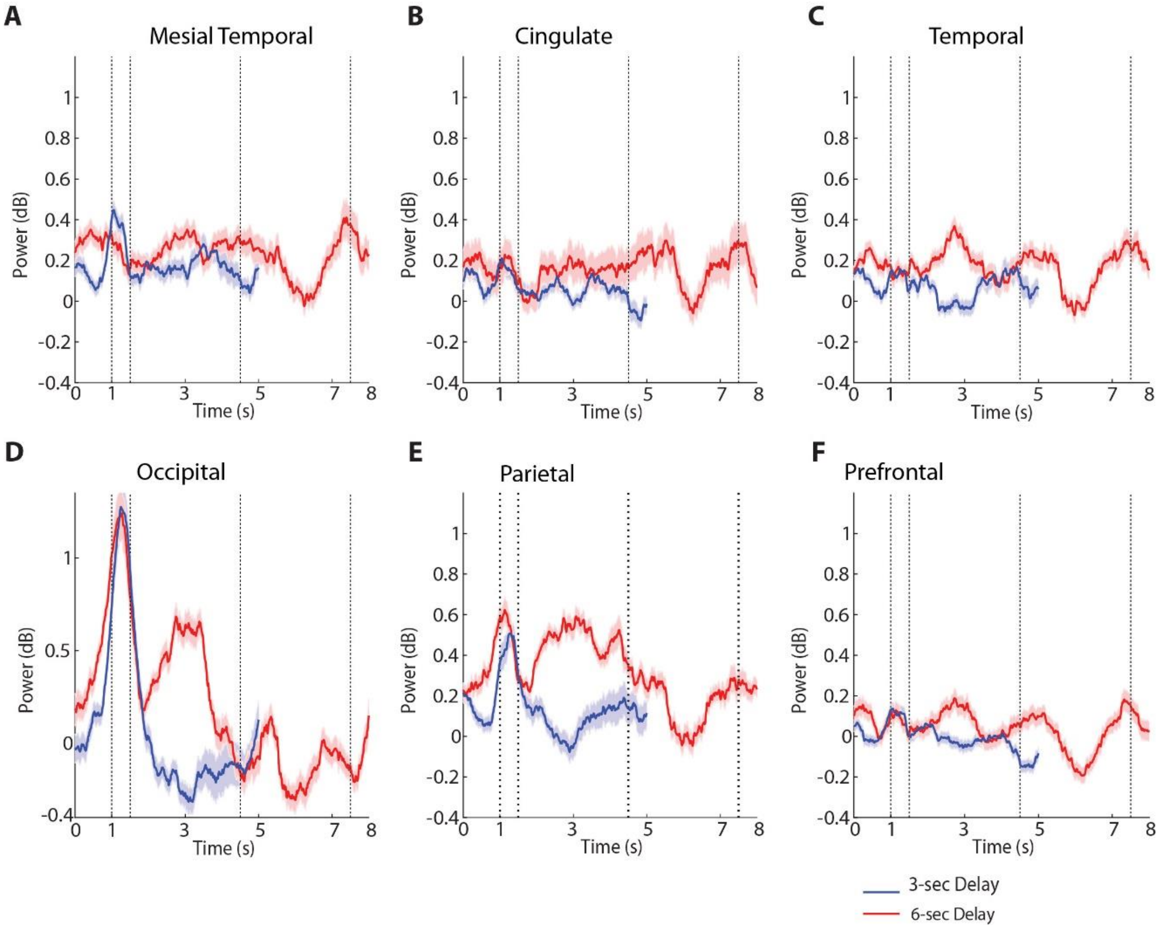

Figure S4, related to Figure 5. High-gamma power for different delay durations. Time resolved induced LFP power in the high-gamma range (100-150 Hz) for the two versions of the spatial working memory task, involving 3 s and 6 s delay periods. **A.** Mesial Temporal regions (n=172 electrode contacts common in 3 s and 6 s delay periods). **B.** Cingulate regions (n=77 contacts). **C.** Temporal regions (n=342 contacts). **D.** Occipital regions (n=37 contacts). **E.** Parietal Regions (n=109 contacts). **F.** Prefrontal regions (n=253 contacts).
